# Influence of Tyrosyl-DNA Phosphodiesterase 1 Inhibitor on the Proapoptotic and Genotoxic Effects of Anticancer Agent Topotecan

**DOI:** 10.1134/S1607672922700077

**Published:** 2023-01-18

**Authors:** A. A. Chepanova, A. L. Zakharenko, N. S. Dyrkheeva, I. A. Chernyshova, O. D. Zakharova, E. S. Ilina, O. A. Luzina, N. F. Salakhutdinov, O. I. Lavrik

**Affiliations:** 1 Institute of Chemical Biology and Fundamental Medicine, Siberian Branch of the Russian Academy of Sciences, Novosibirsk, Russia; 2 Vorozhtsov Novosibirsk Institute of Organic Chemistry, Siberian Branch of the Russian Academy of Sciences, Novosibirsk, Russia

**Keywords:** tyrosyl-DNA phosphodiesterase1, Tdp1 inhibitor, topotecan, DNA repair

## Abstract

To date, various strategies have been proposed to increase the efficiency of cancer therapy. It is known that the action of DNA repair system can determine the resistance of cancer cells to DNA-damaging chemotherapy and radiotherapy, and one of these ways to increase therapeutic efficiency is the search for inhibitors of enzymes of the DNA repair system. Inhibition of the DNA repair enzyme tyrosyl-DNA phosphodiesterase1 (Tdp1) leads to an increase in the effectiveness of the topoisomerase 1 (Top1) inhibitor, the anticancer drug topotecan. Covalent complexes Top1-DNA, which are normally short-lived and are not a threat to the cell, are stabilized under the influence of topotecan and lead to cell death. Tdp1 eliminates such stabilized complexes and thus weaken the effect of topotecan therapy. We have previously shown that the use of the usnic acid hydrazonothiazole derivative **OL9-119** in combination with topotecan increased the antitumor and antimetastatic efficacy of the latter in a mouse model of Lewis lung carcinoma. In this work, it was shown that the combined use of topotecan and Tdp1 inhibitor, the hydrazonothiazole derivative of usnic acid **OL9-119**, leads to an increase in the DNA-damaging effect of topotecan which is used in the clinic for the treatment of cancer. The study of the proapoptotic effect of the compound **OL9-119** showed that the compound itself does not induce apoptosis, but increases the proapoptotic effect of topotecan. The results of the study could be used to improve the effectiveness of anticancer therapy and/or to reduce the therapeutic dose of topotecan and, therefore, the severity of side effects.

Tyrosyl-DNA phosphodiesterase 1 (Tdp1) is a DNA repair enzyme that is responsible for the removal of various endogenous and exogenous adducts from the 3' end of DNA [1]. The most significant 3'-adduct is a covalent adduct of another enzyme, topoisomerase 1 (Top1) with DNA, which is formed during catalysis between the tyrosine residue 721 of Top1 (in humans) and the 3'-end of DNA in a single-strand break [2]. Top1 regulates DNA supercoiling during replication, recombination, and other processes, and this break in one of the DNA strands is necessary to change the topology of the double helix. The covalent complex Top1-DNA is normally short-lived, but can be stabilized by various agents, including anticancer drugs based on camptothecin, topotecan and irinotecan [3]. These camptothecin derivatives bind to the Top1-DNA complex, preventing the religation of DNA and the release of Top1 from the complex, which leads to the stabilization of a single-strand break and can lead to cell death due to blocking of replication, transcription, and other processes [4]. Tdp1 participates in the elimination of such “stuck” complexes, thereby reducing the therapeutic efficacy of topotecan and irinotecan. There is a lot of experimental evidence that a deficiency in Tdp1 activity in cells or organisms leads to hypersensitivity to Top1 inhibitors [5–9]. And vice versa, overexpression of Tdp1 in tumor cells leads to a decrease in sensitivity to Top1 inhibitors [9, 10]. Based on the above, Tdp1 is considered as one of the factors of tumor resistance to therapy with Top1 inhibitors and as a suitable target for accompanying therapy, designed to increase the effectiveness of the main therapy with Top1 inhibitors, including topotecan [11]. Topotecan and irinotecan are selective inhibitors of Top1, that have been used clinically for the last 20 years, including for the treatment of metastatic colorectal and ovarian cancer, small cell lung cancer, cervical carcinoma, and pancreatic adenocarcinoma [12]. Top1 inhibitors cause cell death by apoptosis [12].

Previously, our team discovered a Tdp1 inhibitor, the usnic acid derivative **OL9-119** (laboratory code), capable of sensitizing the cytotoxic effect of topotecan in vitro and its antitumor and antimetastatic effects in vivo (compound **20d** in [13]). We also showed that the combined administration of topotecan and **OL9-119** led to the development of destructive processes in the tumor that were expressed in the appearance of cells in which lipid droplets occupied almost the entire cytoplasm, as well as in an increased accumulation of cellular detritus, that was absent in the samples after the administration of each compound separately [14]. In this work, we studied the level of accumulation of DNA damage in the presence of **OL9-119** alone and in combination with topotecan, as well as the effect of this compound on the proapoptotic effect of topotecan.

To study the effect of **OL9-119** on the accumulation of DNA damages under the action of topotecan, a culture of HeLa cells (cervical cancer) was used. The amount of damage was assessed using the DNA comet method under alkaline conditions [15]. The method is widely used to assess the genotoxic and carcinogenic activity of various agents. The time of incubation of cells with preparations was 1 hour and was chosen on the basis of preliminary experiments (data not shown).

To study the effect of **OL9-119** on topotecan-induced DNA damage accumulation, two concentrations of **OL9-119** were chosen: 5 μM and 20 μM based on its CC_50_ (semi-toxic concentration). The CC_50_ value for the **OL9-119** compound on the HeLa cell line was 9.7 μM, i.e. the first concentration was about 2 times lower than the CC_50_, the second was about 2 times higher. The results obtained are shown in Fig. 1. Control samples of HeLa cells, incubated with 1% DMSO for 1 h, contained a small amount of damaged DNA: 3.8%. When cells were incubated with **OL9-119** at 5 and 20 µM concentrations, the amount of damaged DNA increased to 8.6 and 9.7%, respectively (Fig. 1, Table 1), which was significantly different (p-value < 0.05) from cells incubated with 1% DMSO. Incubation of cells with topotecan at 5 μM concentration (in the presence of 1% DMSO) resulted in an increase in the amount of damaged DNA up to 14.2%.

**Fig. 1.**
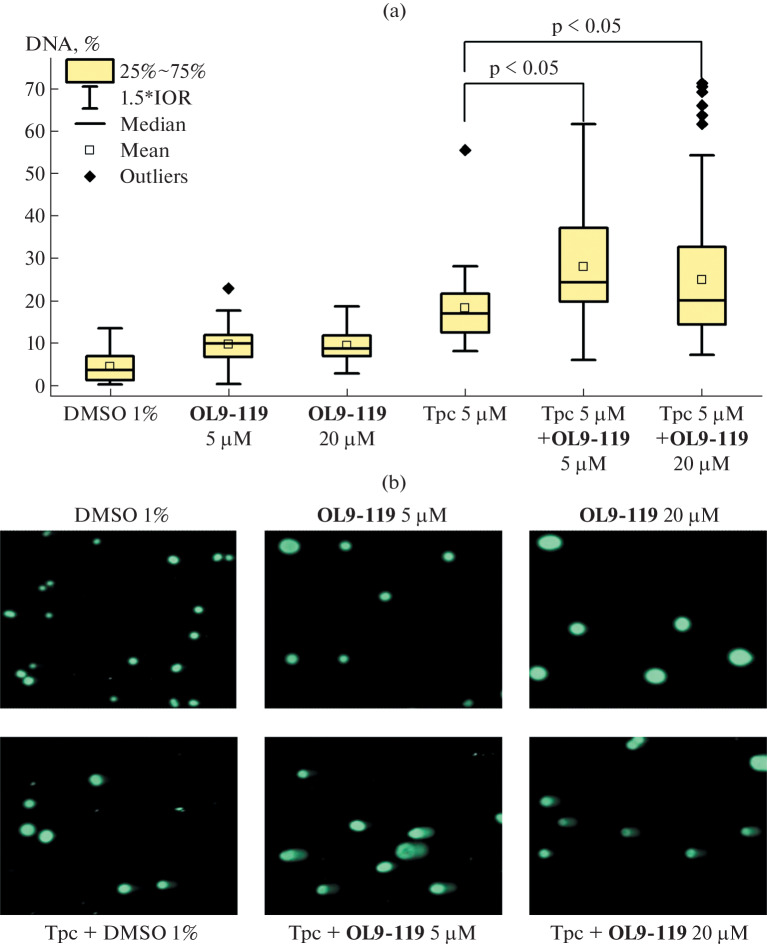
The compound **OL9-119** increases the amount of DNA damages caused by topotecan. (a) Statistical data of experiments on the study of the effect of the compound **OL9-119** at various concentrations on the action of topotecan. (b) Examples of images of DNA comets obtained using fluorescence microscopy.

**Table 1.** Mean amount of DNA damage caused by **OL9-119** alone and in combination with topotecan

**Group**	**Mean amount** **of DNA in tail, %**
No treatment control	3.8 ± 3.2
DMSO 1%	3.9 ± 3.3
**OL9-119** 5 μM	8.7 ± 4.4
**OL9-119** 20 μM	9.8 ± 4.6
Tpc 5 μM	14.2 ± 8.2
Tpc + **OL9-119** 5 μM	28 ± 12
Tpc + **OL9-119** 20 μM	26 ± 15

In the case of the combined use of topotecan and **OL9-119**, the amount of damaged DNA increased significantly compared with the use of topotecan alone from 14.2 to 28%, while did not depend on Tdp1 inhibitor concentration. The amount of DNA damage caused by co-administration of topotecan with **OL9-119** was higher with **OL9-119** at 5 μM (27.9%) than with **OL9-119** at 20 μM (25.5%).

Thus, it was shown that the combined use of topotecan with the Tdp1 inhibitor **OL9-119** enhances the DNA-damaging effect of topotecan. This can be used to increase the effectiveness of anticancer therapy and/or to reduce the dose of topotecan and hence the severity of side effects from the drug action.

One of the problems in the treatment of oncological diseases is the nonspecific high toxicity of anticancer drugs, which is caused, among other things, by the necrotic pathway of cell death. A side effect of the mechanism of action of such drugs is the development of an inflammatory reactions and intoxication. The preferred mechanisms of action on tumor cells, in contrast to necrosis, include the induction of apoptosis, a natural way of cell death [16]. Many of the clinically used anti-cancer drugs cause cell death by apoptosis, including camptothecin and its derivatives irinotecan and topotecan [12].

The type of cell death during the combined use of **OL9-119** and topotecan was studied by flow cytometry with cell staining with Annexin V-FITC and propidium iodide (PI). The combined use of Annexin V and PI allows cells to be divided into living cells (AnV–/PI–), early apoptotic cells (AnV+/PI–), late  apoptotic cells (AnV+/PI+), and necrotic cells (AnV–/PI+) [17].

To analyze the proapoptotic effect of **OL9-119**, two concentrations of this compound, 5 and 20 μM, were chosen. The CC_50_ value of the **OL9-119** compound on the MCF-7 cell line was determined to be 9.3 μM, the first concentration was about 2 times lower than the CC_50_, the second concentration was about 2 times higher. Two concentrations of topotecan were also chosen, 2 and 5 μM. The CC_50_ value of topotecan for the MCF-7 cell line was about 5 μM, the first concentration was 2.5 times lower than CC_50_, and the second was equal to CC_50_. The time of incubation of cells with compounds was chosen based on the development of morphological changes in the cells, after 4 and 8 h at these concentrations of **OL9-119** and topotecan, no morphological changes were observed. After 24 h, many cells with deformed contours were observed detached from the bottom (data not shown). Based on this, the incubation time was chosen to be 14 h.

Control cells incubated with 1% DMSO for 14 hours contained 75% living cells, 4% cells in the early apoptosis, 13.4% cells in the late apoptosis, and 5.8% cells in the necrosis (Fig. 2, Table 2). When cells were incubated with topotecan at 2 and 5 μM concentrations, the population of cells in the early apoptosis remained virtually unchanged compared to the control group of cells. When cells were incubated with topotecan at 2 μM concentration, the population of cells in the late apoptosis increased to 26%, which was two times higher than in the control group of cells. When cells were incubated with topotecan at 5 μM concentration, the population of late apoptotic cells increased to 37.3%, which was three times higher than in the control group of cells. Also, the population of necrosis cells increased up to 13% (p-value <0.05) when cells were incubated with 5 μM topotecan, which was twice higher than in the control group.

**Fig. 2.**
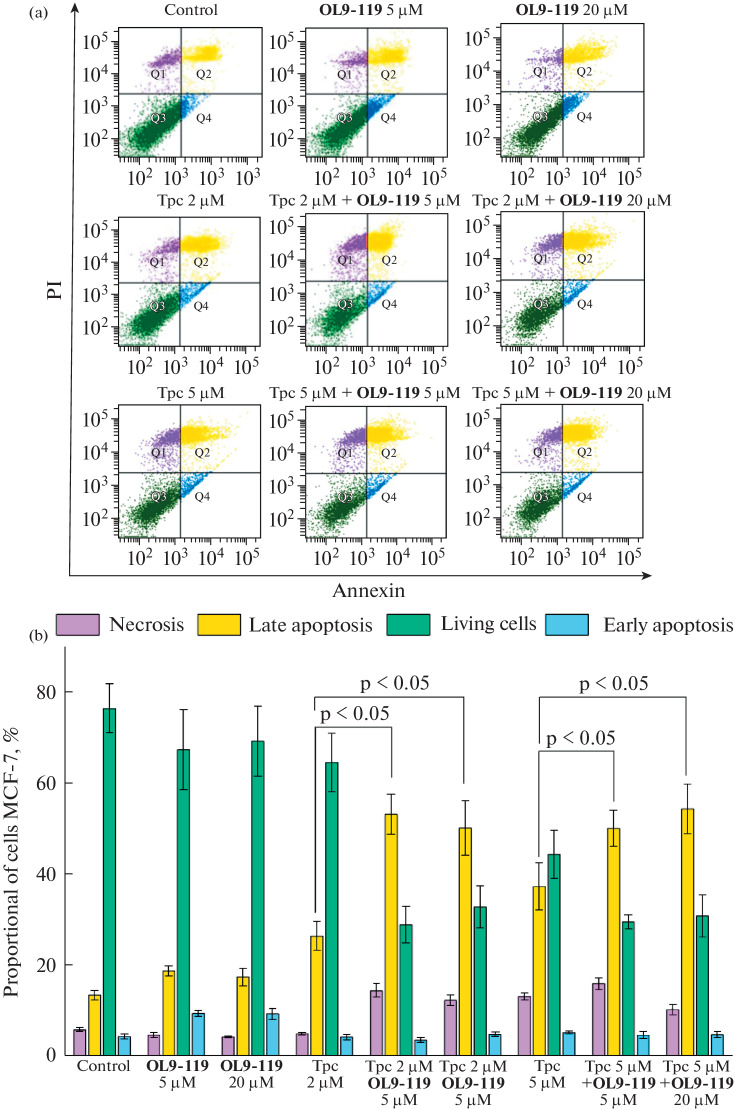
Compound **OL9-119** increases the rate of apoptosis in MCF-7 cells induced by topotecan. Cells were incubated for 14 hours with topotecan at 2 and 5 μM concentrations, Tdp1 inhibitor at 5 and 20 μM concentrations, and their combination, stained with FITC-annexin V/PI, and the level of apoptosis in cell populations was analyzed using flow cytometry. (a) Distribution of cell populations by staining with FITC-annexin V/PI. Q1 – (purple color) necrotic cell population, Q2—(yellow color) late apoptotic cell population, Q3 (green color)—live cells, Q4 (blue color)—early apoptotic cell population. (b) Quantitative analysis of the level of apoptosis in cells treated with Tpc, **OL9-119** and their combination.

**Table 2.** Percentage of cells at various stages of cell death induced by **OL9-119** alone or in combination with topotecan

**Group**	**Q1 (necrosis)**	**Q2 (late apoptosis)**	**Q3 (living cells)**	**Q4 (early apoptosis)**
No treatment control	5.8 ± 0.4	13.4 ± 1.07	76.4 ± 5.3	4.3 ± 0.7
**OL9-119** 5 μM	4.6 ± 0.6	18.7 ± 1.1	67.3 ± 8.8	9.4 ± 0.6
**OL9-119** 20 μM	4.2 ± 0.2	17.4 ± 2	69.2 ± 7.6	9.3 ± 1.2
Tpc 2 μM	4.9 ± 0.3	26.4 ± 3.2	64.5 ± 6.4	4.2 ± 0.6
Tpc 5 μM	13.1 ± 0.8	37.3 ± 5.2	44.3 ± 5.3	5.2 ± 0.3
Tpc 2 μM + **OL9-119** 5 μM	14.4 ± 1.4	53.1 ± 4.4	28.9 ± 4.0	3.5 ± 0.5
Tpc 2 μM + **OL9-119** 20 μM	12.3 ± 1.1	50.1 ± 6.0	32.8 ± 4.6	4.8 ± 0.5
Tpc 5 μM + **OL9-119** 5 μM	15.9 ± 1.3	50.0 ± 4	29.5 ± 1.5	4.6 ± 0.8
Tpc 5 μM + **OL9-119** 20 μM	10.2 ± 1.1	54.3 ± 5.4	30.8 ± 4.6	4.7 ± 0.6

Incubation of cells with **OL9-119** at 5 µM and 20 µM concentrations resulted in an increase in the population of cells in early apoptosis up to 9%, which was 5% more than in the control group. The population of cells late apoptosis also increased by 5% compared with the control group and was 18.7% for **OL9-119** at 5 μM concentration and 17.4% for **OL9-119** at 20 μM concentration. The population of necrotic cells was comparable to the control group when cells were incubated with **OL9-119** at 5 and 20 μM concentrations. Therefore, an increase in the concentration of the Tdp1 inhibitor did not lead to a significant increase in cell death.

As a result of the combined use of 2 μM topotecan and the UA derivative **OL9-119** at 5 and 20 μM concentrations the population of cells in the late apoptosis increased to 53 and 50%, respectively. This was significantly higher compared to only topotecan (2 μM). The growth of the cell population at the late apoptosis was 26% upon co-incubation of 2 μM topotecan and **OL9-119** at 5 μM concentration, and 23% when co-incubated with 2 μM topotecan and 20 μM **OL9-119**. With an increase in the concentration of topotecan to 5 μM in the presence of **OL9-119** at 5 and 20 μM concentrations the population of cells at late apoptosis increased to 50 and 54.3%, respectively.

Population growth compared with 5 μM topotecan alone was 13% with co-incubation of 5 μM topotecan and **OL9-119** at 5 μM and 17% with co-incubation of 5 μM topotecan and **OL9-119** at 20 μM. It is worth noting, that the late apoptosis occurs when the cell membrane becomes permeable, it is characterized by DNA fragmentation and is an irreversible phase of programmed cell death.

Thus, an increase in the fraction of late apoptotic cells under the action of **OL9-119** allows us to conclude that the combined use of topotecan with a Tdp1 inhibitor increases the efficacy of topotecan in the induction of apoptosis and allows to use it at lower concentrations.
